# Effects of Multi-Generational Soft Diet Consumption on Mouse Craniofacial Morphology

**DOI:** 10.3389/fphys.2020.00783

**Published:** 2020-07-10

**Authors:** Mohamed G. Hassan, Harjot Kaler, Bin Zhang, Timothy C. Cox, Nathan Young, Andrew H. Jheon

**Affiliations:** ^1^ Department of Orthodontics, Faculty of Oral and Dental Medicine, South Valley University, Qena, Egypt; ^2^ Program in Craniofacial Biology, University of California, San Francisco, San Francisco, CA, United States; ^3^ Department of Oral and Maxillofacial Surgery, Guanghua School of Stomatology, Hospital of Stomatology, Sun Yat-sen University, Guangzhou, China; ^4^ Guangdong Provincial Key Laboratory of Stomatology, Sun Yat-sen University, Guangzhou, China; ^5^ Center for Developmental Biology and Regenerative Medicine, Seattle Children’s Research Institute, Seattle, WA, United States; ^6^ Department of Orthopaedic Surgery, University of California, San Francisco, San Francisco, CA, United States; ^7^ Divisions of Craniofacial Anomalies and Orthodontics, University of California, San Francisco, San Francisco, CA, United States

**Keywords:** soft diet, craniofacial morphology, multi-generation, mouse, geometric morphometrics, cranium, mandible

## Abstract

Variations in craniofacial morphology may arise as a result of adaptation to different environmental factors such as soft diet (SD), which lessens functional masticatory load. Prior studies have shown that changes in the masticatory muscle function associated with a switch to short-term SD led to changes in craniofacial morphology and alveolar bone architecture. However, the long-term effects of SD and the associated adaptive changes in craniofacial shape are unclear. Our novel study set out to profile prospective skull changes in mice fed with SDs over multiple generations using three-dimensional (3D) geometric morphometric analysis (GMA). Our results revealed that short-term SD consumption led to a significant decrease in craniofacial size, along with numerous shape changes. Long-term SD consumption over 15 continuous generations was not associated with changes in craniofacial size; however, shape analysis revealed mice with shortened crania and mandibles in the anteroposterior dimension, as well as relative widening in the transverse dimension compared to the average shape of all mice analyzed in our study. Moreover, changes in shape and size associated with different functional loads appeared to be independent – shape changes persisted after diets were switched for one generation, whereas size decreased after one generation and then returned to baseline size. Our study is the first to study the role of prolonged, multi-generational SD consumption in the determination of craniofacial size and shape.

## Introduction

Can exclusive and prolonged consumption of soft diets (SDs) alter our and our offsprings’ craniofacial morphology? Human skulls have undergone morphological transformations over centuries with changes being most pronounced in areas associated with masticatory muscle attachments (Jantz, 2001; Godde, 2015; Manthey et al., 2017). Changes in human skull form (shape and size) have been associated with transition to softer diets, from ancestral hunter-gatherer culture to that of agriculture and farming (Larsen, 2006; Pinhasi et al., 2015). Furthermore, transition to softer diets following the Industrial Revolution has been hypothesized to be one of the primary reasons for adaptive changes to craniofacial structures (Larsen, 2006; von Cramon-Taubadel, 2011; Rando et al., 2014). Neo-Lamarckian evolution suggests that the environment can directly alter phenotype and this acquired trait is passed down in a heritable manner through environmental epigenetics and epigenetic transgenerational inheritance (Skinner, 2015). Furthermore, parental diets have been shown previously to potentially influence the presence of a wide range of craniofacial deformities in offsprings (Hassan et al., 2019; Wang et al., 2019).

SD consumption over a single generation results in craniofacial structural and morphological changes, which characterize functional adaptation of the somatic cells and tissues in the skull due to functional load differences (Mavropoulos et al., 2010, 2014; Chen et al., 2011; Dias et al., 2011; Utreja et al., 2016; Ödman et al., 2019). Prior studies have clearly demonstrated that short-term SD decreases total craniofacial bone volume, bone thickness, alveolar bone trabecular volume, and subchondral condylar bone volume (Odman et al., 2008; Mavropoulos et al., 2010; Anderson et al., 2014). Moreover, mandibles showed shorter alveolar processes, smaller coronoid and gonial processes, and extruded molars compared to control mice fed with a hard diet (HD) (Odman et al., 2008; Mavropoulos et al., 2010; Anderson et al., 2014). Notably, all of these experiments were conducted over a single generation using different two-dimensional (2D) measurements (Kiliaridis et al., 1985; Bresin, 2001; Mavropoulos et al., 2004; Enomoto et al., 2010; Guerreiro et al., 2013).

Morphological changes due to prolonged, multi-generational SD, if diet is indeed an important factor, are clearly not isolated to a single generation. Furthermore, 2D morphometric analysis is insufficient to capture variation across complex craniofacial structures as the essential units of morphology are shape and size, which are difficult to accurately assess with linear and angular measurements. The limitations of 2D analysis may be overcome by utilizing micro-computed tomography (microCT) with three-dimensional (3D) geometric morphometric analysis (GMA) (Bouvier and Hylander, 1982; Sylvester, 2013). 3D GMA utilizes the placement of landmarks to analyze 3D shape variation between specimens in an unbiased manner and is central to evaluating functional and evolutionary hypotheses (Slice, 2005; Freudenthaler et al., 2017). However, landmark-based GMA still suffers from limitations in the characterization of organismal form. More complex structures such as the condyle and glenoid fossa are more challenging to accurately quantify using traditional landmarks, as it can be challenging to identify sufficient landmarks to represent 3D shape. Thus, semi-/pseudo-landmarks were introduced as additional tools to characterize complex 3D shapes (Mitteroecker et al., 2004; Gunz et al., 2005). These approaches enhanced the representation of complex shapes, by densely sampling the regions that may not have many discrete points of geometric homology within or between them but represent homologous structures across specimens. Thus, the main advantage of semi-landmarks is the ability to readily represent homologous curves and surfaces by sets of points to establish geometric homology between different subjects or specimens (Bardua et al., 2019; Goswami et al., 2019).

The aim of our novel study was to identify the effects of prolonged, multi-generational SD consumption on mouse craniofacial morphology using 3D GMA. Toward this end, we fed mice HD (normal mouse chow) vs. SD over 15 continuous generations and performed 3D GMA and 2D linear morphometrics on mouse crania and mandibles. Semi-landmarking was utilized on the condyle. Short-term SD resulted in a decrease in mouse craniofacial size. Prolonged SD did not affect the size of the cranium and mandible but shape changes, such as widening in the transverse dimension and decrease in the anteroposterior dimension of both the cranium and mandible were noted.

## Materials and Methods

### Animals and Experimental Design

**Figure 1:**
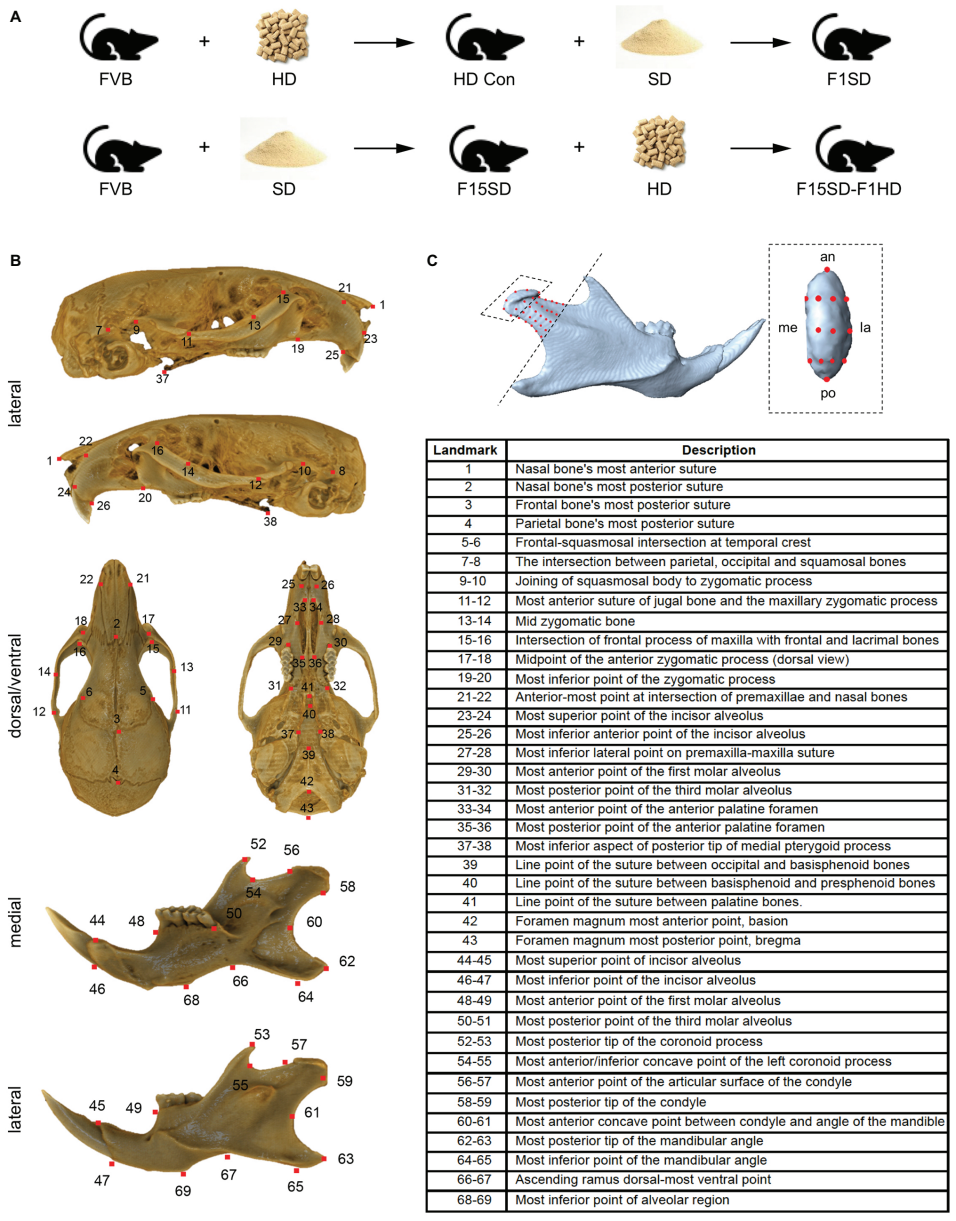
ContinuedFIGURE 1Mouse skull and landmarks. **(A)** Experimental design. FVB mice were placed on soft diet (SD) or hard diet/normal chow (HD) for 15 continuous generations (F15SD and HD Con, respectively). Subsequently, HD Con and F15SD mice were switched to SD and HD for a single generation, respectively (F1SD and F15SD-F1HD). **(B)** A segmented cranium and mandible. *Red dots* indicate all landmarks. Lateral, dorsal, ventral, and medial. **(C)** A segmented hemi-mandible in lateral view showing semi-landmarks (*red dots*) of the condylar head (*black dashed box*) and neck proximo-dorsal to the set border (*black dashed line*). **(C’)** Magnified view of the condylar head. *Red dots* indicate all landmarks. an, anterior; di, distal; la, lateral; me, medial; po, posterior; pr, proximal.

FVB mice (Charles River), which are inbred and generate a relatively high number of pups (6–8) per litter, were utilized for these studies. At the initiation of our study, pregnant FVB mice at embryonic (E) day 0.5 (i.e., vaginal plug was observed) were maintained on HD (normal mice chow) or switched to SD (PicoLab 5058, LabDiet, Deans Animal Feeds, Redwood City, CA, USA). This diet regimen was maintained continuously for 15 (F15SD) generations. With each new generation, non-littermate males and females (two mating pairs) were randomly selected (runts were excluded) and paired for mating to continue the HD or SD mouse lines. HD Con and F15SD mice were sacrificed at the same time. We then switched HD Con and F15SD mice to SD (F1SD) and HD (F15SD-F1HD), respectively, for one generation. F1SD and F15SD-F1HD mice were sacrificed at the same time. HD and SD are equivalent in nutrient content, differing only in hardness (Deans Animal Feeds). Sample sizes in these groups were HD Con (*N* = 12; 6 males and 6 females), F15SD (*N* = 12; 6 males and 6 females), F1SD group (*N* = 8; 4 males and 4 females), and F15SD-F1HD (*N* = 10; 5 males and 5 females) (Figure 1A). All mice were weaned at 21 days. At 6 weeks of age, mice were weighed, sacrificed, and fixed in 4% paraformaldehyde at 4°C for 48 h. All aspects of animal care and experiments were approved by the Institutional Animal Care and Use Committee (IACUC) at the University of California San Francisco (UCSF) and performed under animal research protocol number AN164201.

### Image Processing and Landmark Data Collection Protocol

Fixed mouse heads were imaged by microCT using a SkyScan 1076 MicroCT at the Small Animal Tomographic Analysis Facility (SANTA) located at Seattle Children’s Research Institute, United States. Specimens were scanned at 17.2 micron resolution (55 kV, 150 mA, 0.5 mm Al filter). Reconstructions were generated using NRecon (Version 1.6.9.4) with consistent thresholding parameters, and converted to 3D volumes. Skull segmentation and the generation of bony surfaces from microCT data were performed with Avizo Lite (version 9.1.1).

### Landmarking

We established three sets of landmarks: Set 1 included 13 paired bilateral landmarks that characterized the morphology of the mandible, Set 2 included 43 landmarks that characterized the morphology of the cranium (Figure 1B), and Set 3 included 50 semi-landmarks distributed across the right condyle of all specimens – nine of these points were placed along the condylar head and neck along an imaginary line passing from the tip of the coronoid process to the gonial angle (Figure 1C). We applied semi-landmarking techniques to quantify 3D condylar shapes since semi-landmarks can significantly improve the characterization of complex structures (Bardua et al., 2019).

### Traditional Morphometrics Data

Traditional morphometrics employed standard measurements routinely used in analyses of mouse skull variations. We took the following distances measured from the cranium and mandible: skull length (SL); skull width (SW); neurocranium width (NW); mandible length (ML), bi-condylar width (BCW), bi-gonial width (BGW), and intermolar width (IMW) (Supplementary Figure S1). All measurements were taken using Landmark software (Wiley et al., 2005). Mandible length measurements were performed on right hemi-mandibles.

### Principal Component and Canonical Variate Analyses

Landmark coordinates were exported as text files and imported to MorphoJ software to perform GMA (Klingenberg, 2011). For each set, landmarks were superimposed using generalized Procrustes analysis (GPA) and the resulting Procrustes coordinates were assessed using principal component analysis (PCA) and canonical variate analysis (CVA) (Slice, 2005). These procedures minimize the influence of size and adopt a single orientation for all specimens by shifting the landmark configurations to a common position, scaling them to a standard size, and rotating them until a best fit of corresponding landmarks is achieved. GPA, therefore, locks the effects of scale, translation, and rotation but does not eliminate the allometric shape variation that is related to size (Rohlf, 1996; Slice, 2005).

For this study, variations in craniofacial shape for these samples were assessed using PCA. We carried out PCA using the residuals of multivariate regression of Procrustes coordinates on centroid size to investigate the shape variation independent of size (Darroch and Moismann, 1985; Falsetti et al., 1993). PCA of Procrustes coordinates is based on an eigenvalue decomposition of a covariance matrix, which transforms the Procrustes coordinates into scores along with principal components (PCs). In most cases, the first few PCs explain most of the variance in the dataset. Each observation is scored for each principal axis and the scores of an observation along the principal axes map that observation into the morphospace using MorphoJ (Klingenberg, 2011).

In addition, we carried out CVA to identify shape features that maximize the separation between groups using variances within each group. Changes in the shape of crania and mandibles were displayed visually using the wireframe outlines in MorphoJ compared to the average shape along each canonical variate with the outlines at the extremes. Lastly, we calculated the centroid size (the square root of the sum of squared Euclidean distances from each landmark to their own centroid) for the cranium and mandible. Generally, this measurement is the standard for assessing size in GMA (Le and Kume, 2000; Schwarze et al., 2019).

### Statistical Analysis

Descriptive statistics were calculated for linear measurements and centroid sizes. For significance tests, we used one-way ANOVA. Graphpad Prism (Version 8.0.2) was used to perform tests and graphics. To determine whether shape differences were statistically significant, values of *p* were also calculated using Procrustes ANOVA and Mahalanobis distances (Supplementary Table S1). To determine inter-observer landmark reproducibility, two observers (MGH and HK) independently located the landmarks on 10 randomly selected samples (Supplementary Figure S2). A 10,000 round permutation test was performed on the Procrustes distance between the observers’ landmarked samples, testing for mean overall shape differences between them. For linear measurements, intra-observer reliability was analyzed using the paired-*t* test and Bland-Altman method (Supplementary Figure S3). The main observer (MGH) took a second set of measurements (seven randomly selected samples) 1 week after the first set to confirm the absence of intra-observer error.

## Results

Our study analyzed four groups of mice: (1) HD Con mice, (2) F15SD mice, (3) F1SD mice, and (4) F15SD-F1HD mice (Figure 1A). The descriptive statistics of the variables used in this study, centroid size, and linear measurements are presented (Table 1).

**Table 1:** Descriptive statistics of all variables and comparisons between groups.

Measured	Groups	Mean	Std. deviation	Std. error of mean
Skull (Cranium) centroid size	HD Con	45350	1549	447.1
	F1SD	43434	590.8	208.9
	F15SD	45114	607.6	175.4
	F15SD-F1HD	42949	907.2	286.9
Skull (Cranial) length (SL)	HD Con	22783	746.8	215.6
	F1SD	21696	227.9	80.56
	F15SD	22598	325.9	94.07
	F15SD-F1HD	21501	505.0	159.7
Skull (Cranial) width (SW)	HD Con	11565	360.8	104.1
	F1SD	11104	242.9	85.90
	F15SD	11546	164.4	47.45
	F15SD-F1HD	11042	178.2	56.35
Neurocranium width (NW)	HD Con	10772	145.2	41.92
	F1SD	10305	109.9	38.85
	F15SD	10710	86.19	24.88
	F15SD-F1HD	10381	117.2	37.06
Mandible centroid size	HD Con	26699	911.7	263.2
	F1SD	25571	464.1	164.1
	F15SD	26785	238.0	68.71
	F15SD-F1HD	25342	475.5	150.4
Mandible length (ML)	HD Con	11457	444.4	128.3
	F1SD	10749	118.7	41.96
	F15SD	11571	158.8	45.84
	F15SD-F1HD	10756	294.6	93.17
Bi-condylar width (BCW)	HD Con	10322	222.1	64.10
	F1SD	9876	253.2	89.53
	F15SD	10312	188.3	54.36
	F15SD-F1HD	10038	276.2	87.33
Bi-gonial width (BGW)	HD Con	9513	337.6	97.45
	F1SD	9097	488.2	172.6
	F15SD	9537	313.3	90.44
	F15SD-F1HD	9098	459.5	145.3
Intermolar width (IMW)	HD Con	4038	204.6	59.05
	F1SD	3689	63.23	22.36
	F15SD	3771	71.74	20.71
	F15SD-F1HD	3590	64.15	20.29

### Effects of SD on Cranial Morphology

**Figure 2:**
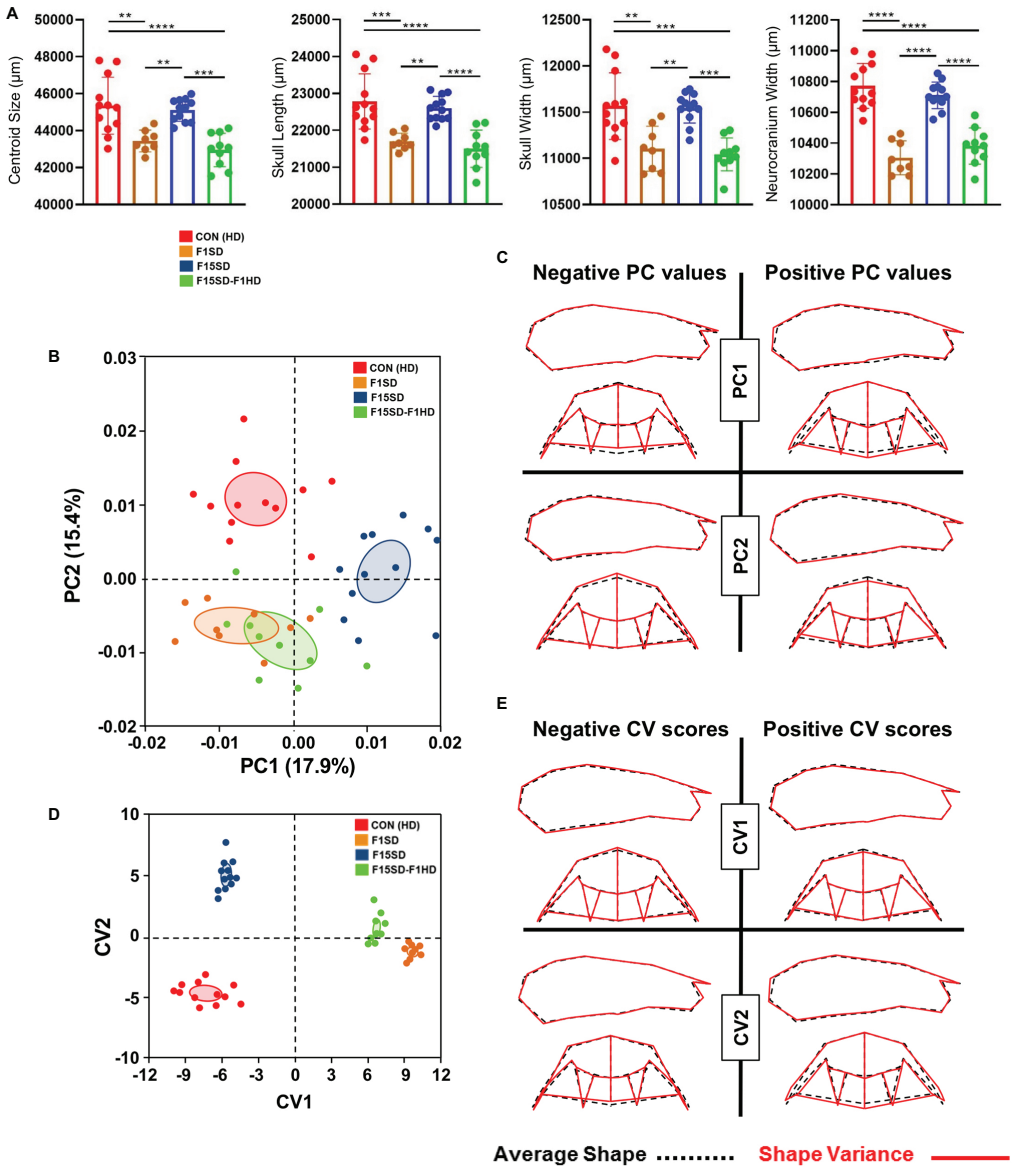
Morphological changes of the cranium in 6-week old mice. **(A)** F1SD and F15SD-F1HD cranium centroid sizes and linear measurements were smaller than HD Con and F15SD. *Post hoc* comparisons using the Tukey’s test with one-way ANOVA. Data represents the means ± SD. **(B)** Principal component analysis (PCA) of cranium shape: HD Con (red), F1SD (brown), F15SD (blue), and F15SD-F1HD (green). The ellipses represent the confidence range of the means of each group. **(C)** Wireframe illustrations demonstrate shape changes associated with the extreme positive and negative principal component (PC)1 and PC2 values in shape (red line), and average shapes (dotted black line). **(D)** Canonical variate analysis (CVA) shows clear separation between HD Con and F15SD mice confirming shape differences between the two groups. **(E)** Wireframe illustrations demonstrate shape changes associated with the extreme positive and negative canonical variates (CV)1 and CV2 values in shape (red line) and average shapes (dotted black line). **p < 0.005; ***p < 0.0005; ****p < 0.0001.

A one-way ANOVA was conducted to illustrate the effect of SD on craniofacial morphology. There was a significant difference in mean centroid size [*F*(3,38) = 14.25, *p* < 0.0001] between the four groups. *Post hoc* comparisons using the Tukey’s test were carried out. Switching diets for a single generation significantly decreased cranial size in F1SD and F15SD-F1HD mice (Figure 2A; Table 2). Notably, there were no significant differences in the cranial measurements between HD Con and F15SD mice (Figure 2A; Table 2).

**Table 2:** Pairwise comparisons from one-way ANOVA between the means of the size and linear measurements in the four diet groups.

Measured	Groups		Mean difference (A–B)	SE of difference	*p* value
	Group A	Group B			
Skull (Cranial) centroid size	HD Con	F1SD	1916	470.0	0.0012^**^
		F15SD	235.7	420.4	0.9431
		F15SD-F1HD	2401	440.9	<0.0001^****^
	F15SD	F1SD	-1680	470.0	0.0052^**^
		F15SD-F1HD	2165	440.9	0.0001^***^
Skull (Cranial) length (SL)	HD Con	F1SD	1087	233.7	0.0002^***^
		F15SD	185.1	209.0	0.8124
		F15SD-F1HD	1282	219.2	<0.0001^****^
	F15SD	F1SD	-901.7	233.7	0.0023^**^
		F15SD-F1HD	1097	219.2	<0.0001^****^
Skull (Cranial) width (SW)	HD Con	F1SD	460.8	115.4	0.0016^**^
		F15SD	19.32	103.2	0.9976
		F15SD-F1HD	522.8	108.2	0.0001^***^
	F15SD	F1SD	-441.5	115.4	0.0025^**^
		F15SD-F1HD	503.5	108.2	0.0002^***^
Neurocranium width (NW)	HD Con	F1SD	467.4	53.48	<0.0001^****^
		F15SD	61.76	47.84	0.5742
		F15SD-F1HD	391.1	50.17	<0.0001^****^
	F15SD	F1SD	-405.6	53.48	<0.0001^****^
		F15SD-F1HD	329.3	50.17	<0.0001^****^
Mandible centroid size	HD Con	F1SD	1128	270.1	0.0009^***^
		F15SD	-85.44	241.6	0.9846
		F15SD-F1HD	1357	253.4	<0.0001^****^
	F15SD	F1SD	-1214	270.1	0.0004^***^
		F15SD-F1HD	1443	253.4	<0.0001^****^
Mandible length (ML)	HD Con	F1SD	708.2	135.1	<0.0001^****^
		F15SD	-114.1	120.8	0.7814
		F15SD-F1HD	700.4	126.7	<0.0001^****^
	F15SD	F1SD	-822.3	135.1	<0.0001^****^
		F15SD-F1HD	814.5	126.7	<0.0001^****^
Bi-condylar width (BCW)	HD Con	F1SD	446.4	106.5	0.0009^***^
		F15SD	9.938	95.23	0.9996
		F15SD-F1HD	284.3	99.88	0.0343^*^
	F15SD	F1SD	-436.4	106.5	0.0012^**^
		F15SD-F1HD	274.4	99.88	0.0434^*^
Bi-gonial width (BGW)	HD Con	F1SD	415.5	179.9	0.1136
		F15SD	-24.09	160.9	0.9988
		F15SD-F1HD	414.2	168.7	0.0839
	F15SD	F1SD	-439.6	179.9	0.0858
		F15SD-F1HD	438.3	168.7	0.0613
Intermolar width (IMW)	HD Con	F1SD	348.7	56.49	<0.0001^****^
		F15SD	266.6	50.52	<0.0001^****^
		F15SD-F1HD	448.0	52.99	<0.0001^***^
	F15SD	F1SD	-82.15	56.49	0.4744
		F15SD-F1HD	181.5	52.99	0.0078^**^

*p < 0.05; **p < 0.005; ***p < 0.0005; ****p < 0.0001.

The shape of the mouse crania differed significantly (Procrustes ANOVA, *F* = 14.72, *p* < 0.0001; Supplementary Table S1). When the shape of the cranium was considered (i.e., without the effects of centroid size), the majority of the variation was accounted for by PC1 and PC2 (17.9 and 15.4%, respectively, Figure 2B, Supplementary Figure S4). F15SD mice possessed a shorter midface were wider transversely due to outwardly displaced zygomatic arches and taller vertically due to raised posterior cranial vaults (Figures 2B,C). Conversely, HD Con mice were associated with forward displacement of the midface and were narrower transversely due to inwardly displaced zygomatic arches and shorter vertically due to depressed posterior cranial vaults (Figures 2B,C).

CVA revealed aspects of cranial shape variation capable of discriminating among the four groups, following adjustment for centroid size (Figure 2D). The first and second canonical variates (CV1 and CV2) accounted for 96% of the variation (data not shown). CV1 accounted for more than 75% of the observed shape variation. CV1 positive scores were associated with smaller crania, depressed posterior cranial vaults, anterior displaced midface, and narrowed transverse widths due to inwardly displaced zygomatic arches (Figure 2E). Accounting for 20% of the shape variation, CV2 separated the four groups as well, with HD Con mice at one end, F15SD mice at the other, and F1SD and F15SD-F1HD mice in between (Figure 2D). Clusters with positive CV2 values such as F15SD mice possessed wider zygomatic arches and shortened midface in the anteroposterior dimension (Figure 2E). Conversely, HD Con mice were clustered at negative CV2 values and possessed narrowed zygomatic arches, raised posterior cranial vaults, and longer alveolar ridge relative to the average shape (Figure 2E). F1SD and F15SD-F1HD mice were clustered close together in between HD Con and F15SD mice (Figure 2D).

### Effects of SD on Mandibular Morphology

**Figure 3:**
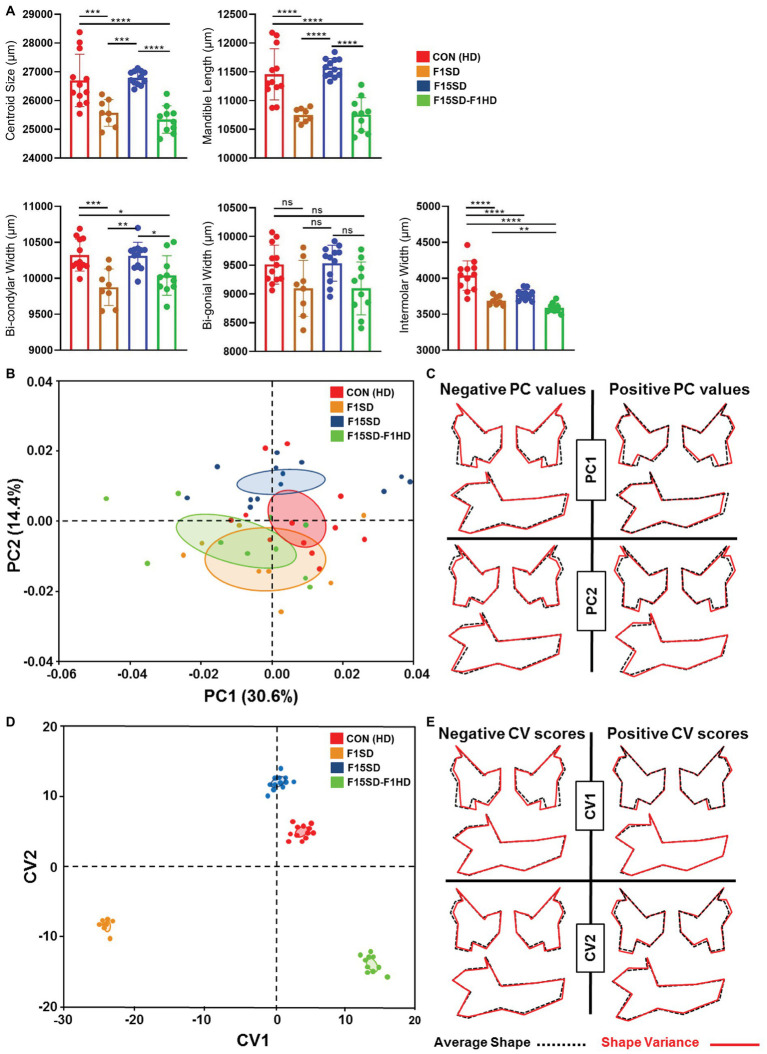
Continued FIGURE 3 | Morphological changes of the mandible in 6-week old mice. **(A)** F1SD and F15SD-F1HD mandible centroid sizes and linear measurements were smaller than HD Con and F15SD. *Post hoc* comparisons using the Tukey test with one-way ANOVA **(A)**. Data represents the means ± SD. **(B)** PCA of mandibular shape: HD Con (red), F1SD (brown), F15SD (blue), and F15SD-F1HD (green). The ellipses represent the confidence range of the means of each group. **(C)** Wireframe illustrations demonstrate shape changes associated with the extreme positive and negative PC1 and PC2 values in shape (red line) and average shapes (dotted black line). **(D)** CVA shows clear separation between HD Con and F15SD mice confirming shape differences between the two groups. **(E)** Wireframe illustrations demonstrate shape changes associated with the extreme positive and negative CV1 and CV2 values in shape (red line) and average shapes (dotted black line). *p < 0.05; **p < 0.005; ***p < 0.0005; ****p < 0.0001.

Similar to the cranium, there were statistically significant differences in the mean centroid size [*F*(3,38) = 16.76, *p* < 0.0001] between groups as determined by one-way ANOVA. The biggest difference in size was again observed with switching of diets in a single generation, F1SD and F15SD-F1HD mice. A Tukey’s *post hoc* test revealed that the mandible centroid size, as well as linear measurements of F1SD and F15SD-F1HD mandibles were significantly lower than HD Con and F15SD mandibles (Figure 3A; Table 2). There were no significant differences in BGW between the groups but the IMW was significantly lower in F1SD, F15SD, and F15SD-F1HD compared to HD Con mandibles.

The shape of the mouse mandibles differed significantly (Procrustes ANOVA, *F* = 16.76, *p* < 0.0001; Supplementary Table S1). PCA of allometry-free shape data showed clear separation of HD Con and F15SD mandibles. The first two PCs of the PCA accounted for 45% of the shape variance (Figure 3B, Supplementary Figure S4). There were differences in mandible shape that discriminated between the groups: F1SD and F15SD-F1HD mice possessed longer mandibular bodies, wider and shorter mandibular rami, posteriorly displaced coronoid processes, and shallow mandibular notches (Figure 3C). Notably, F15SD mice had shorter mandibular bodies, narrower and elongated mandibular rami, anteriorly displaced coronoid processes, and a deep mandibular notch (Figure 3C).

CVA revealed distinct mandibular shape variation among the four different groups (Figure 3D). CV1 and CV2 were associated with 91% of the variation (data not shown). Clusters with lower values of CV1, such as F1SD and F15SD-F1HD mice, presented with longer mandibles and smaller transverse dimensions due to inwardly displaced mandibular condyles and gonial angles (Figure 3E). Furthermore, clusters with positive values of CV2, such as F15SD mice, possessed shorter mandibles and elongated and narrower rami (Figure 3E).

### Effects of SD on Condyle Morphology

**Figure 4:**
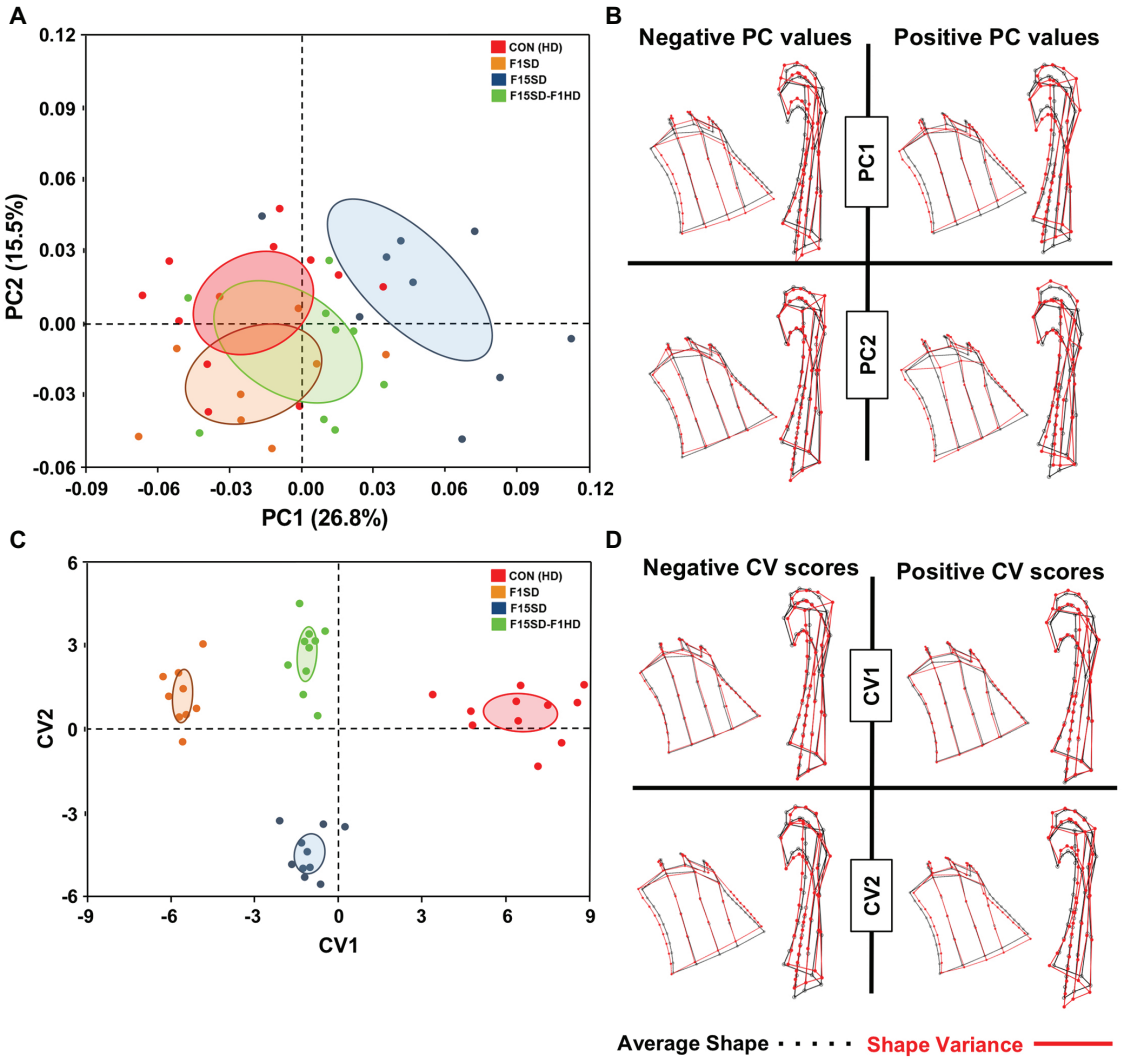
Morphological changes of the condyle in 6-week old mice. **(A)** PCA of condylar shape by population (using the set of 50 semi-landmarks on the right condyle): HD Con (red), F1SD (brown), F15SD (blue), and F15SD-F1HD (green). The ellipses represent the confidence range of the means of each group. **(B)** Wireframe illustrations demonstrate shape changes associated with the extreme positive and negative PC1 and PC2 values in shape (red line), and average shapes (dotted black line). **(C)** CVA shows clear separation between HD Con and F15SD mice confirming shape differences between the two groups. **(D)** Wireframe illustrations demonstrate shape changes associated with the extreme positive and negative CV1 and CV2 values in shape (red line) and average shapes (dotted black line).

Using semi-landmarks and GMA to analyze condyle morphology, multivariate regression of Procrustes coordinates on centroid size showed that 12.3% of shape variation was due to static allometry in condyle morphometry between the four groups. The shape of the mouse condyles differed significantly (Procrustes ANOVA, *F* = 2.98, *p* < 0.0001; Supplementary Table S1). PC1 and PC2 were associated with 42% of shape variation in the mandibular condyle of all specimens (Figure 4A; Supplementary Figure S4). Clusters with higher values of PC1 represented F15SD mice with wider condylar necks in the anteroposterior dimension and outwardly displaced condylar heads, in comparison to HD Con mice that were located at the negative end values of PC1 (Figure 4B). F1SD and F15SD-F1HD mice were clustered in between HD Con and F15SD mice. CVA was consistent with PCA outcomes and clearly distinguished the four groups (Figure 4C). CV1 and CV2 were associated with 86% of the variation (data not shown). Positive CV1 values such as that observed for control mice possessed inwardly displaced condyles (Figure 4D). Clusters with negative CV2 values such as in F15SD mice presented with wider condylar necks and outwardly displaced condylar heads (Figure 4D).

## Discussion

Our study, to our knowledge, is the first to investigate changes in mouse craniofacial morphology due to prolonged, multi-generational exposure to SD using 3D GMA. Prior studies using animal models have analyzed craniofacial adaptation to SD and load reduction in a single generation using 2D linear measurements (Corruccini and Beecher, 1982; Kiliaridis et al., 1985; Dias et al., 2011; Denes et al., 2013, 2018; Anderson et al., 2014; Goto et al., 2015; Kono et al., 2017). Mice are preferred models because of the use of isogenic strains, tight environmental control of multiple specimens, relatively short gestation periods, and extensive information from prior genetic and environmental studies (Fish, 2016; Liu, 2016; Van Otterloo et al., 2016).

### Short-Term Effects of SD on Craniofacial Morphology

Our results showed that short-term SD (F1SD mice) significantly decreased craniofacial size (Figures 2, 3). These results are consistent with studies analyzing the effects of low masticatory function on craniofacial structures in one generation using 2D linear measurements (Kiliaridis, 2006; Odman et al., 2008; Guerreiro et al., 2013; Hichijo et al., 2015; Fujita and Maki, 2018; Ödman et al., 2019). Interestingly, one study demonstrated that SD has no effect on the rat cranial length and width despite using the same linear measurements (Katsaros et al., 2002).

With respect to size-free shape changes, our results were partially consistent with prior studies (Kiliaridis et al., 1985; Kono et al., 2017). Our F1SD mice possessed transverse widening of zygomatic/temporal regions, flattening of the neurocrania, less convex angular processes, shallow antegonial notches, inward tipping of the condylar processes, and lengthening of the mandibular bodies. Prior studies largely showed flattening of the neurocrania and less convex angular processes but not our other findings (Kiliaridis et al., 1985; Kono et al., 2017). The differences in our results and prior studies may be due to differences in experimental design. In prior studies such as by Katsaros et al. (2002), rats were given SD at weaning (28 days) for a 4-week duration. In our study, F1SD mice were exposed to SD beginning at E0.5 (parents were given on SD) until 6 weeks old. Although F1SD mice did not directly eat SD from E0.5 to ~21 days of age, they developed in cages with SD and were indirectly exposed to SD through the parents, and it is unclear the exact age that F1SD would begin to eat SD. Thus, it is interesting to posit whether indirect exposure to SD *via* parents or early exposure to SD may influence offspring morphology.

The condyles and condylar processes of F1SD mice showed decreases in the anteroposterior dimension, findings that are in agreement with prior studies in which masticatory hypofunction decreased condylar dimensions (Kiliaridis et al., 1999; Scheidegger et al., 2018). It should be noted that condylar heights were not analyzed in our study because the lower boundary of the semi-landmarks was based on an imaginary line passing through the tip of the coronoid process and the angle of the mandible, which are anatomical sites prone to functional load adaptations.

### Long-Term Effects of SD on Craniofacial Morphology

Our experimental design exposed mice to 15 continuous generations of SD to observe long-term morphological effects. Our design would eliminate the confounding influence of transient weight loss presumably due to adaptation to a new diet, which has been noted upon switching to SD (Kiliaridis et al., 1985). The fact that no differences were recorded in mean cranium and mandible centroid sizes nor in the linear measurements of the craniofacial structures between HD Con and F15SD mice demonstrated that long-term SD had no impact on skull size. Moreover, we did not observe any differences in weights between HD Con and F15SD male and female mice (Supplementary Figure S5). Our experimental design is the likely reason that changes in craniofacial morphology in F15SD mice differ from all prior single generation studies. F15SD mice possessed oval neurocrania with depressed posterior cranial vaults, outward displaced zygomatic arches, and posteriorly displaced maxillary alveolar bones. F15SD mandibles were shortened in the anteroposterior dimension and were associated with increases in the transverse dimension, decreases in mandibular alveolar bone heights, and accentuated anti-gonial notches. F15SD condyles presented with increases in the anteroposterior dimension and outward displacement, which were coordinated with transverse widening of zygomatic/temporal bones and mandibles. The lateral displacement of the zygomatico-temporal region was coordinated with lateral displacement of the mandibular posterior region and condyle.

Interestingly, the changes in F15SD skull morphology were similar in one prior study in pigs fed SD (Larsson et al., 2005). The pigs were fed SD at weaning (5 weeks old) until 22 months of age, the longest duration of time that an animal had been fed SD in a single generation study. The pigs showed significantly wider arches and fewer posterior crossbites compared to HD-fed animals, similar to that observed in our F15SD mice.

## Conclusion

To our knowledge, our study is the first to analyze mice on prolonged, multi-generational SD to determine the effects of diet consistency on craniofacial form. Our results suggest that changes in craniofacial shape and size in response to decreased functional loads may be independent. Short-term SD resulted in smaller cranial and mandibular sizes likely due to transitional adaptation of mice to new diets. Prolonged SD did not affect the size of the cranium and mandible but altered the shape. Shape changes associated with prolonged SD consumption included widening in the transverse dimension, as well as decreases in the anteroposterior dimension of both the cranium and mandible. The lateral displacement of the zygomatico-temporal region of the cranium was coordinated with lateral displacement of the posterior mandible including the condyle.

## Data Availability Statement

All datasets generated for this study are included in the article/ Supplementary Material and upon request.

## Ethics Statement

The animal study was reviewed and approved by Institutional Animal Care and Use Committee (IACUC) at UCSF and performed under animal research protocol number AN164201.

## Author Contributions

MH and AJ designed the study. BZ maintained the mice. MH and HK performed the landmarking. TC performed the micro-computed tomography. MH conducted the geometric morphometric analysis. NY reviewed the analysis. MH and AJ wrote the manuscript. All authors contributed to the article and approved the submitted version.

### Conflict of Interest

The authors declare that the research was conducted in the absence of any commercial or financial relationships that could be construed as a potential conflict of interest.

## References

[ref1] Anderson P. S. L. Renaud S. Rayfield E. J. (2014). Adaptive plasticity in the mouse mandible. BMC Evol. Biol. 14:85. 10.1186/1471-2148-14-85, PMID: 24742055 PMC4002541

[ref2] Bardua C. Felice R. N. Watanabe A. Fabre A. -C. Goswami A. (2019). A practical guide to sliding and surface semilandmarks in morphometric analyses. Integr. Comp. Biol. 1, 1–34. 10.1093/iob/obz016 PMC7780474 33791531

[ref3] Bouvier M. Hylander W. L. (1982). The effect of dietary consistency on morphology of the mandibular condylar cartilage in young macaques (*Macaca mulatta*). Prog. Clin. Biol. Res. 101, 569–579. PMID: 7156160

[ref4] Bresin A. (2001). Effects of masticatory muscle function and bite-raising on mandibular morphology in the growing rat. Swed. Dent. J. Suppl. 150, 1–49. PMID: 11803646

[ref5] Chen J. Sobue T. Utreja A. Kalajzic Z. Xu M. Kilts T.. (2011). Sex differences in chondrocyte maturation in the mandibular condyle from a decreased occlusal loading model. Calcif. Tissue Int. 89, 123–129. 10.1007/s00223-011-9498-9, PMID: 21597908 PMC3298998

[ref6] Corruccini R. S. Beecher R. M. (1982). Occlusal variation related to soft diet in a nonhuman primate. Science 218, 74–76. 10.1126/science.7123221, PMID: 7123221

[ref7] Darroch J. N. Mosimann J. E. (1985). Canonical and principal components of shape. Biometrika 72, 241–252. 10.1093/biomet/72.2.241

[ref8] Denes B. J. Lazzarotto B. Bresin A. Kiliaridis S. (2018). Effect of different masticatory functional demands on the 3D mandibular condyle morphology of growing rats using posterior bite-blocks. Eur. J. Orthod. 40, 312–316. 10.1093/ejo/cjx072, PMID: 29040460

[ref9] Denes B. J. Mavropoulos A. Bresin A. Kiliaridis S. (2013). Influence of masticatory hypofunction on the alveolar bone and the molar periodontal ligament space in the rat maxilla. Eur. J. Oral Sci. 121, 532–537. 10.1111/eos.12092, PMID: 24206071

[ref10] Dias G. J. Cook R. B. Mirhosseini M. (2011). Influence of food consistency on growth and morphology of the mandibular condyle. Clin. Anat. 24, 590–598. 10.1002/ca.21122, PMID: 21647960

[ref11] Enomoto A. Watahiki J. Yamaguchi T. Irie T. Tachikawa T. Maki K. (2010). Effects of mastication on mandibular growth evaluated by microcomputed tomography. Eur. J. Orthod. 32, 66–70. 10.1093/ejo/cjp060, PMID: 19648440

[ref12] Falsetti A. B. Jungers W. L. Colle T. M. (1993). Morphometrics of the callitrichid forelimb: a case study in size and shape. Int. J. Primatol. 14, 551–572. 10.1007/BF02215447

[ref13] Fish J. L. (2016). Developmental mechanisms underlying variation in craniofacial disease and evolution. Dev. Biol. 415, 188–197. 10.1016/j.ydbio.2015.12.019, PMID: 26724698

[ref14] Freudenthaler J. Čelar A. Ritt C. Mitteröcker P. (2017). Geometric morphometrics of different malocclusions in lateral skull radiographs. J. Orofac. Orthop. 78, 11–20. 10.1007/s00056-016-0057-x, PMID: 27796401 PMC5247554

[ref15] Fujita Y. Maki K. (2018). Association of feeding behavior with jaw bone metabolism and tongue pressure. Jpn. Dent. Sci. Rev. 54, 174–182. 10.1016/j.jdsr.2018.05.001 30302136 PMC6175966

[ref16] Godde K. (2015). Secular trends in cranial morphological traits: a socioeconomic perspective of change and sexual dimorphism in north Americans 1849-1960. Ann. Hum. Biol. 42, 253–259. 10.3109/03014460.2014.941399, PMID: 25156659

[ref17] Goswami A. Watanabe A. Felice R. N. Bardua C. Fabre A. -C. Polly P. D. (2019). High-density morphometric analysis of shape and integration: the good, the bad, and the not-really-a-problem. Integr. Comp. Biol. 59, 669–683. 10.1093/icb/icz120, PMID: 31243431 PMC6754122

[ref18] Goto S. Fujita Y. Hotta M. Sugiyama A. Maki K. (2015). Influence of differences in the hardness and calcium content of diets on the growth of craniofacial bone in rats. Angle Orthod. 85, 969–979. 10.2319/102214-765.1, PMID: 25630054 PMC8612051

[ref19] Guerreiro F. d. S. Diniz P. Carvalho P. E. G. Ferreira E. C. Avancini S. R. P. Ferreira-Santos R. I. (2013). Effects of masticatory hypofunction on mandibular morphology, mineral density and basal bone area. Braz. J. Oral Sci. 12, 205–211. 10.1590/S1677-32252013000300010

[ref20] Gunz P. Mitteroecker P. Bookstein F. L. (2005). “Semilandmarks in three dimensions” in Modern morphometrics in physical anthropology. ed. Slice D. E. (New York: Kluwer Academic Publishers-Plenum Publishers), 73–98.

[ref21] Hassan M. G. Vargas R. Zaher A. R. Ismail H. A. Lee C. Cox T. C.. (2019). Altering calcium and phosphorus levels in utero affects adult mouse mandibular morphology. Orthod. Craniofac. Res. 22(Suppl. 1), 113–119. 10.1111/ocr.12269, PMID: 31074150

[ref22] Hichijo N. Tanaka E. Kawai N. van Ruijven L. J. Langenbach G. E. J. (2015). Effects of decreased occlusal loading during growth on the mandibular bone characteristics. PLoS One 10:e0129290. 10.1371/journal.pone.0129290, PMID: 26062027 PMC4465670

[ref23] Jantz R. L. (2001). Cranial change in Americans: 1850–1975. J. Forensic Sci. 46, 784–787. 10.1520/jfs15047j, PMID: 11451056

[ref24] Katsaros C. Berg R. Kiliaridis S. (2002). Influence of masticatory muscle function on transverse skull dimensions in the growing rat. J. Orofac. Orthop. 63, 5–13. 10.1007/s00056-002-9903-0, PMID: 11974452

[ref25] Kiliaridis S. (2006). The importance of masticatory muscle function in dentofacial growth. Semin. Orthod. 12, 110–119. 10.1053/j.sodo.2006.01.004

[ref26] Kiliaridis S. Engström C. Thilander B. (1985). The relationship between masticatory function and craniofacial morphology. I. a cephalometric longitudinal analysis in the growing rat fed a soft diet. Eur. J. Orthod. 7, 273–283. 10.1093/ejo/7.4.273, PMID: 3865789

[ref27] Kiliaridis S. Thilander B. Kjellberg H. Topouzelis N. Zafiriadis A. (1999). Effect of low masticatory function on condylar growth: a morphometric study in the rat. Am. J. Orthod. Dentofacial Orthop. 116, 121–125. 10.1016/S0889-5406(99)70207-6, PMID: 10434083

[ref28] Klingenberg C. P. (2011). MorphoJ: an integrated software package for geometric morphometrics. Mol. Ecol. Resour. 11, 353–357. 10.1111/j.1755-0998.2010.02924.x, PMID: 21429143

[ref29] Kono K. Tanikawa C. Yanagita T. Kamioka H. Yamashiro T. (2017). A novel method to detect 3D mandibular changes related to soft-diet feeding. Front. Physiol. 8:567. 10.3389/fphys.2017.00567, PMID: 28855872 PMC5557733

[ref30] Larsen C. S. (2006). The agricultural revolution as environmental catastrophe: implications for health and lifestyle in the Holocene. Quat. Int. 150, 12–20. 10.1016/j.quaint.2006.01.004

[ref31] Larsson E. Øgaard B. Lindsten R. Holmgren N. Brattberg M. Brattberg L. (2005). Craniofacial and dentofacial development in pigs fed soft and hard diets. Am. J. Orthod. Dentofacial Orthop. 128, 731–739. 10.1016/j.ajodo.2004.09.025, PMID: 16360913

[ref32] Le H. Kume A. (2000). Detection of shape changes in biological features. J. Microsc. 200, 140–147. 10.1046/j.1365-2818.2000.00744.x, PMID: 11106954

[ref34] Liu K. J. (2016). Animal models of craniofacial anomalies. Dev. Biol. 415, 169–170. 10.1016/j.ydbio.2016.06.008, PMID: 27321071

[ref35] Manthey L. Jantz R. L. Bohnert M. Jellinghaus K. (2017). Secular change of sexually dimorphic cranial variables in Euro-Americans and Germans. Int. J. Leg. Med. 131, 1113–1118. 10.1007/s00414-016-1469-2, PMID: 27757580

[ref36] Mavropoulos A. Kiliaridis S. Bresin A. Ammann P. (2004). Effect of different masticatory functional and mechanical demands on the structural adaptation of the mandibular alveolar bone in young growing rats. Bone 35, 191–197. 10.1016/j.bone.2004.03.020, PMID: 15207756

[ref37] Mavropoulos A. Kiliaridis S. Rizzoli R. Ammann P. (2014). Normal masticatory function partially protects the rat mandibular bone from estrogen-deficiency induced osteoporosis. J. Biomech. 47, 2666–2671. 10.1016/j.jbiomech.2014.05.012, PMID: 24925255

[ref38] Mavropoulos A. Odman A. Ammann P. Kiliaridis S. (2010). Rehabilitation of masticatory function improves the alveolar bone architecture of the mandible in adult rats. Bone 47, 687–692. 10.1016/j.bone.2010.06.025, PMID: 20601301

[ref39] Mitteroecker P. Gunz P. Bernhard M. Schaefer K. Bookstein F. L. (2004). Comparison of cranial ontogenetic trajectories among great apes and humans. J. Hum. Evol. 46, 679–697. 10.1016/j.jhevol.2004.03.006, PMID: 15183670

[ref40] Ödman A. Bresin A. Kiliaridis S. (2019). The effect of retraining hypofunctional jaw muscles on the transverse skull dimensions of adult rats. Acta Odontol. Scand. 77, 184–188. 10.1080/00016357.2018.1531437, PMID: 30623708

[ref41] Odman A. Mavropoulos A. Kiliaridis S. (2008). Do masticatory functional changes influence the mandibular morphology in adult rats. Arch. Oral Biol. 53, 1149–1154. 10.1016/j.archoralbio.2008.07.004, PMID: 18721914

[ref42] Pinhasi R. Eshed V. von Cramon-Taubadel N. (2015). Incongruity between affinity patterns based on mandibular and lower dental dimensions following the transition to agriculture in the near East, Anatolia and Europe. PLoS One 10:e0117301. 10.1371/journal.pone.0117301, PMID: 25651540 PMC4317182

[ref43] Rando C. Hillson S. Antoine D. (2014). Changes in mandibular dimensions during the mediaeval to post-mediaeval transition in London: a possible response to decreased masticatory load. Arch. Oral Biol. 59, 73–81. 10.1016/j.archoralbio.2013.10.001, PMID: 24169153

[ref44] Rohlf F. J. (1996). “Morphometric spaces, shape components and the effects of linear transformations” in Advances in morphometrics. eds. Marcus M. Corti C. Loy L. Slice S. (New York: Plenum Press), 131–152.

[ref45] Scheidegger R. Koletsi D. Eliades T. (2018). The impact of dietary consistency on structural craniofacial components: temporomandibular joint/condyle, condylar cartilage, alveolar bone and periodontal ligament. A systematic review and meta-analysis in experimental in vivo research. Arch. Oral Biol. 94, 33–47. 10.1016/j.archoralbio.2018.06.016, PMID: 29957455

[ref46] Schwarze U. Y. Dobsak T. Gruber R. Bookstein F. L. (2019). Anatomical similarity between the sost-knockout mouse and sclerosteosis in humans. Anat. Rec. 10.1002/ar.24318, PMID: [Epub ahead of print]. 31729194 PMC7496997

[ref47] Skinner M. K. (2015). Environmental epigenetics and a unified theory of the molecular aspects of evolution: a Neo-Lamarckian concept that facilitates Neo-Darwinian evolution. Genome Biol. Evol. 7, 1296–1302. 10.1093/gbe/evv073, PMID: 25917417 PMC4453068

[ref48] Slice D. E. (ed.) (2005). “Modern morphometrics” in Modern morphometrics in physical anthropology. (New York: Kluwer Academic Publishers-Plenum Publishers), 1–45.

[ref49] Sylvester A. D. (2013). A geometric morphometric analysis of the medial tibial condyle of African hominids. Anat. Rec. 296, 1518–1525. 10.1002/ar.22762, PMID: 23956043

[ref51] Utreja A. Dyment N. A. Yadav S. Villa M. M. Li Y. Jiang X.. (2016). Cell and matrix response of temporomandibular cartilage to mechanical loading. Osteoarthr. Cartil. 24, 335–344. 10.1016/j.joca.2015.08.010, PMID: 26362410 PMC4757844

[ref52] Van Otterloo E. Williams T. Artinger K. B. (2016). The old and new face of craniofacial research: how animal models inform human craniofacial genetic and clinical data. Dev. Biol. 415, 171–187. 10.1016/j.ydbio.2016.01.017, PMID: 26808208 PMC4914413

[ref53] von Cramon-Taubadel N. (2011). Global human mandibular variation reflects differences in agricultural and hunter-gatherer subsistence strategies. Proc. Natl. Acad. Sci. U. S. A. 108, 19546–19551. 10.1073/pnas.1113050108, PMID: 22106280 PMC3241821

[ref54] Wang Q. Kurosaka H. Kikuchi M. Nakaya A. Trainor P. A. Yamashiro T. (2019). Perturbed development of cranial neural crest cells in association with reduced sonic hedgehog signaling underlies the pathogenesis of retinoic-acid-induced cleft palate. Dis. Model. Mech. 12:dmm040279. 10.1242/dmm.040279, PMID: 31591086 PMC6826016

[ref55] Wiley D. F. Amenta N. Alcantara D. Ghosh D. Kil Y. Delson E. (2005). Evolutionary Morphing (In Proceedings). Minneapolis, Minnesota: *Proceedings of IEEE Visualization* 2005.

