# Supplementary material for: Effects of Multi-Generational Soft Diet Consumption on Mouse Craniofacial Morphology
Source: Front Physiol. 2020 Jul 10;11:783. doi: 10.3389/fphys.2020.00783 (PMC7367031; doi:10.3389/fphys.2020.00783)
Supplement: Supplementary file 1 [file Data_Sheet_1.PDF]

Fig. S1. (A) Landmarking and linear measurements. (B) A snapshot of the Landmark program with loaded specimens.

A

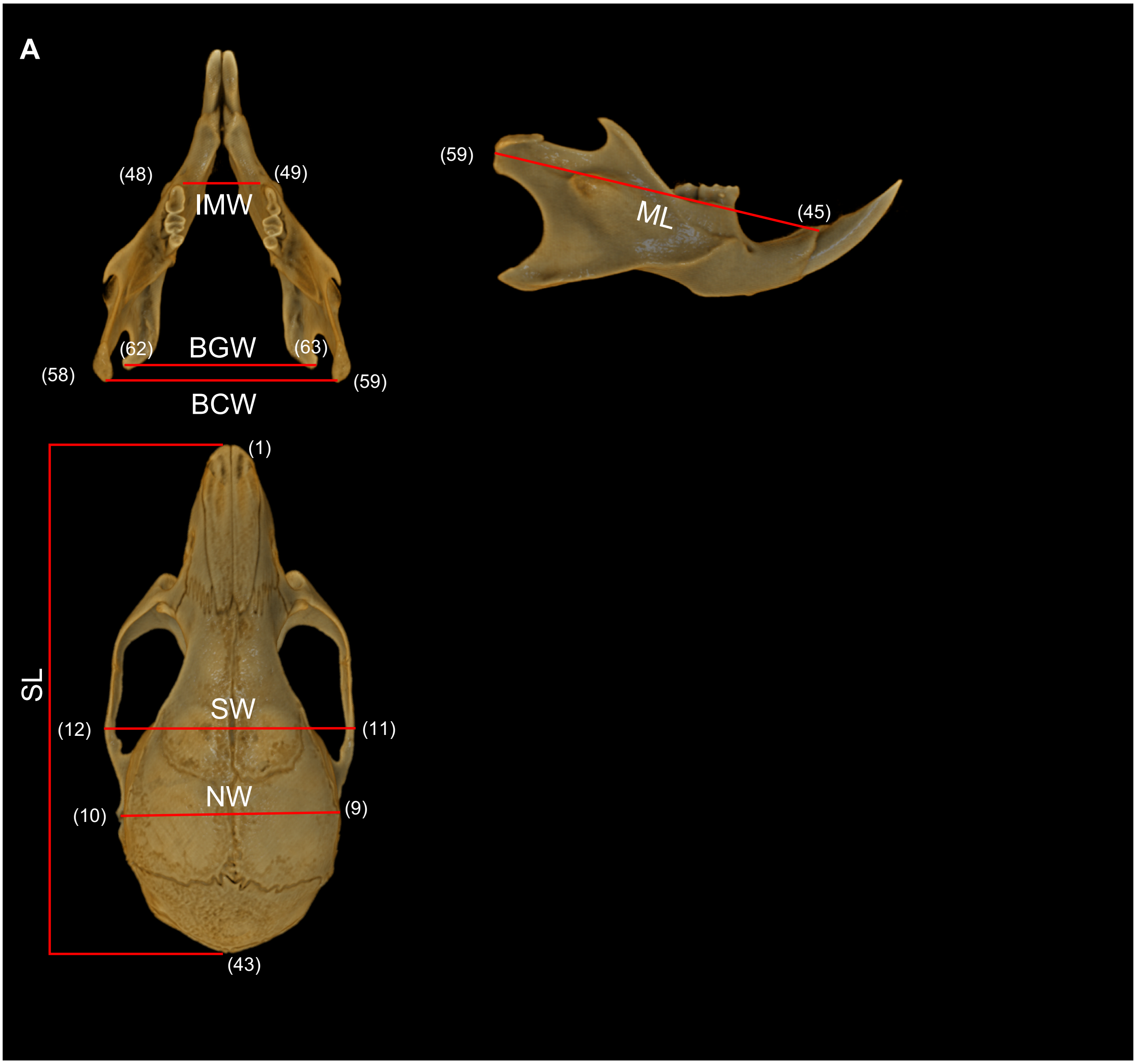

B

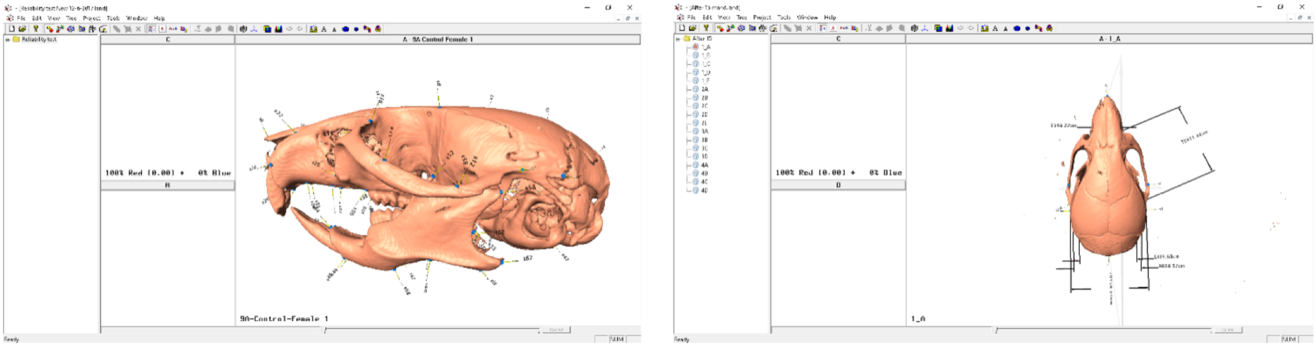

Table S1. Determination of statistically significant shape differences for the mouse cranium, mandible, and condyle.

Procrustes ANOVA

	Effect	SS	MS	*df*	*F*	P-value
**Cranium**	**Centroid Size**	45702003.490723	15234001.16357	3	14.72	< 0.0001
	**Shape**	0.00526248	0.0000269871	195	6.34	<0.0001
**Mandible**	**Centroid Size**	17609239.190990	5869746.396997	3	16.76	<0.0001
	**Shape**	0.00727317	0.0000692683	105	5.49	<0.0001
**Condyle**	**Centroid Size**	1067152.739130	355717.579710	3	2.53	0.0723
	**Shape**	0.05558199	0.0001295618	429	2.98	<0.0001

Sums of squares (SS), mean squares (MS), degrees of freedom (*df*), and Goodall's F statistic (F).

Mahalanobis distances among groups

		Con (HD)	F15SD-F1HD	F15SD
**Cranium**	**F15SD-F1HD**	15.1479*		
	**F15SD**	9.8843*	13.4091*	
	**F1SD**	17.5256*	5.9188*	16.6211*
**Mandible**	**F15SD-F1HD**	23.2906*		
	**F15SD**	14.7058*	29.0896*	
	**F1SD**	31.5735*	38.0982*	32.2284*
**Condyle**	**F15SD-F1HD**	8.9554*		
	**F15SD**	9.3830*	7.3755*	
	**F1SD**	12.2569*	7.2336*	8.0526*

P value <0.0001*

Fig. S2

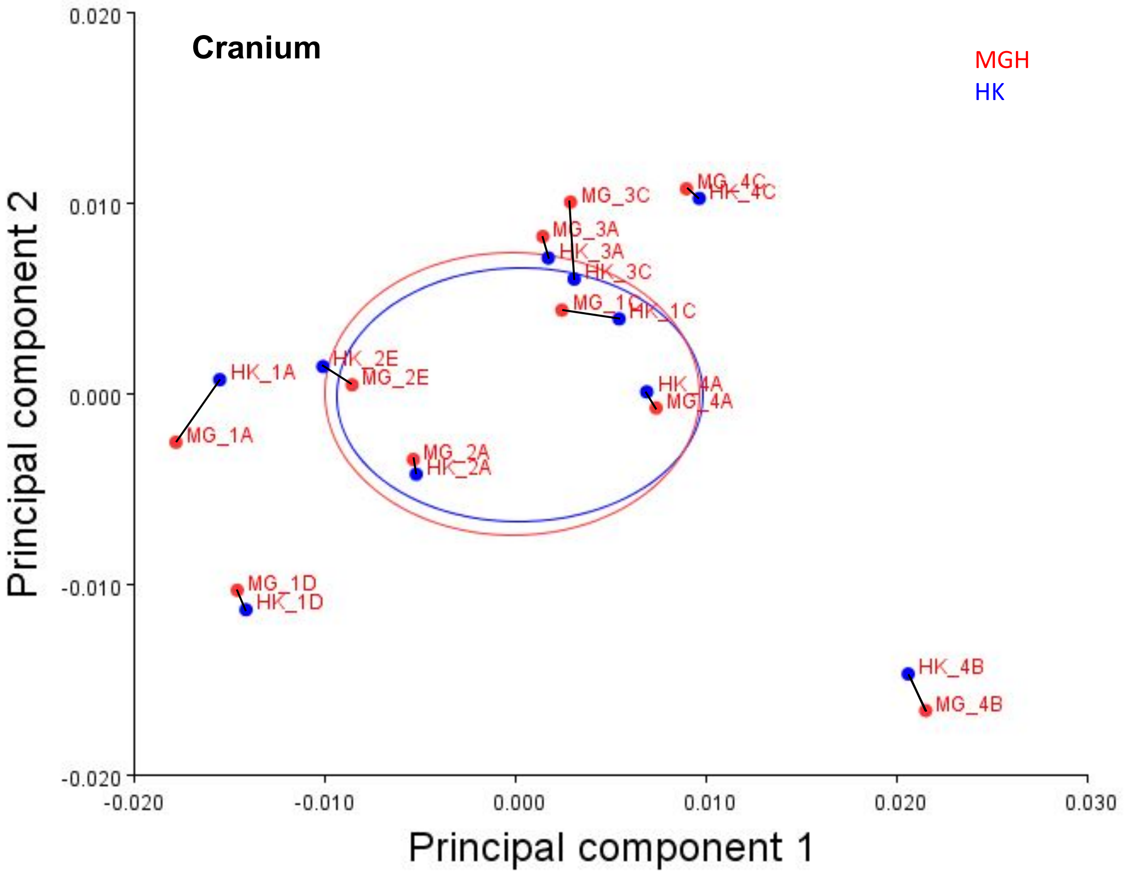

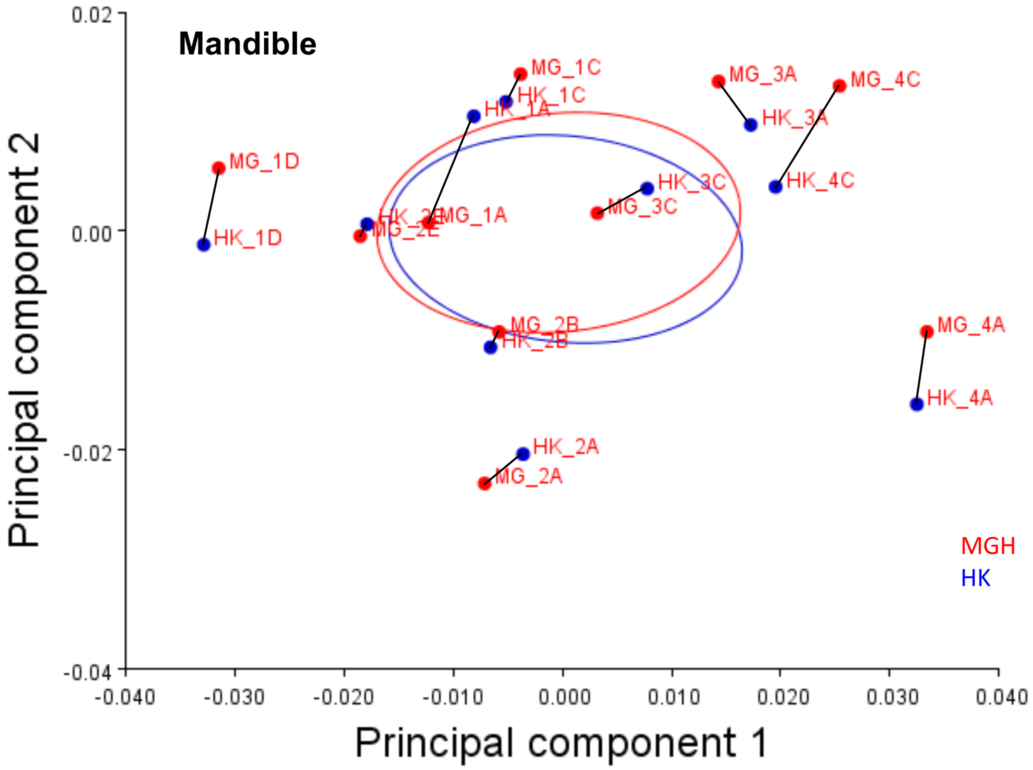

Fig. S2. Example of the inter-reliability test between observers MGH and HK demonstrating the consistency of landmarking in the cranium and mandible.

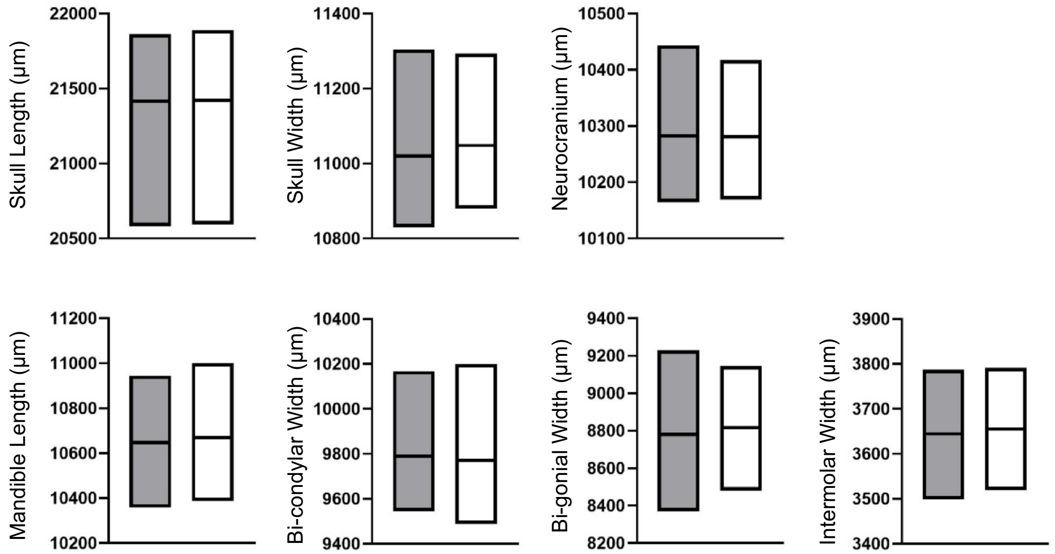

Paired t-test applied to the linear variables demonstrates little or no intra-examiner error.

Score/timing	Number	Mean	SD	P value
**Skull Length (SL)**<br>1st Time<br>2nd Time	7<br>7	21417<br>21423	432.6<br>441.5	0.7669
**Skull Width (SW)**<br>1st Time<br>2nd Time	7<br>7	11021<br>11048	153.8<br>127.4	0.1348
**Neurocranium Width (NW)**<br>1st Time<br>2nd Time	7<br>7	10282<br>10280	102.8<br>91.62	0.9040
**Mandible Length (ML)**<br>1st Time<br>2nd Time	7<br>7	10648<br>10669	206<br>223.8	0.1145
**Bi-condylar Width (BCW)**<br>1st Time<br>2nd Time	7<br>7	9790<br>9771	240.1<br>264.1	0.2666
**Bi-gonial Width (BGW)**<br>1st Time<br>2nd Time	7<br>7	8780<br>8816	340<br>267.9	0.3599
**Intermolar Width (IMW)**<br>1st Time<br>2nd Time	7<br>7	3644<br>3655	101.3<br>103.1	0.0671

Bland-Altman method applied to the linear variables demonstrates little or no intra-examiner error.

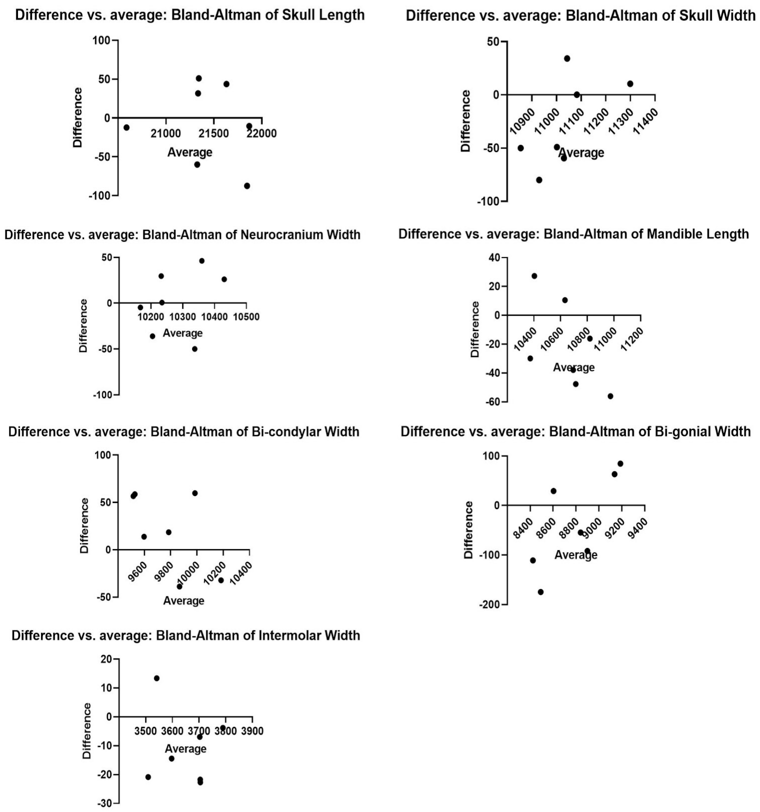

Fig. S3. Paired t-test and Bland-Altman method applied to the linear measurements to evaluate intra-observer reliability.

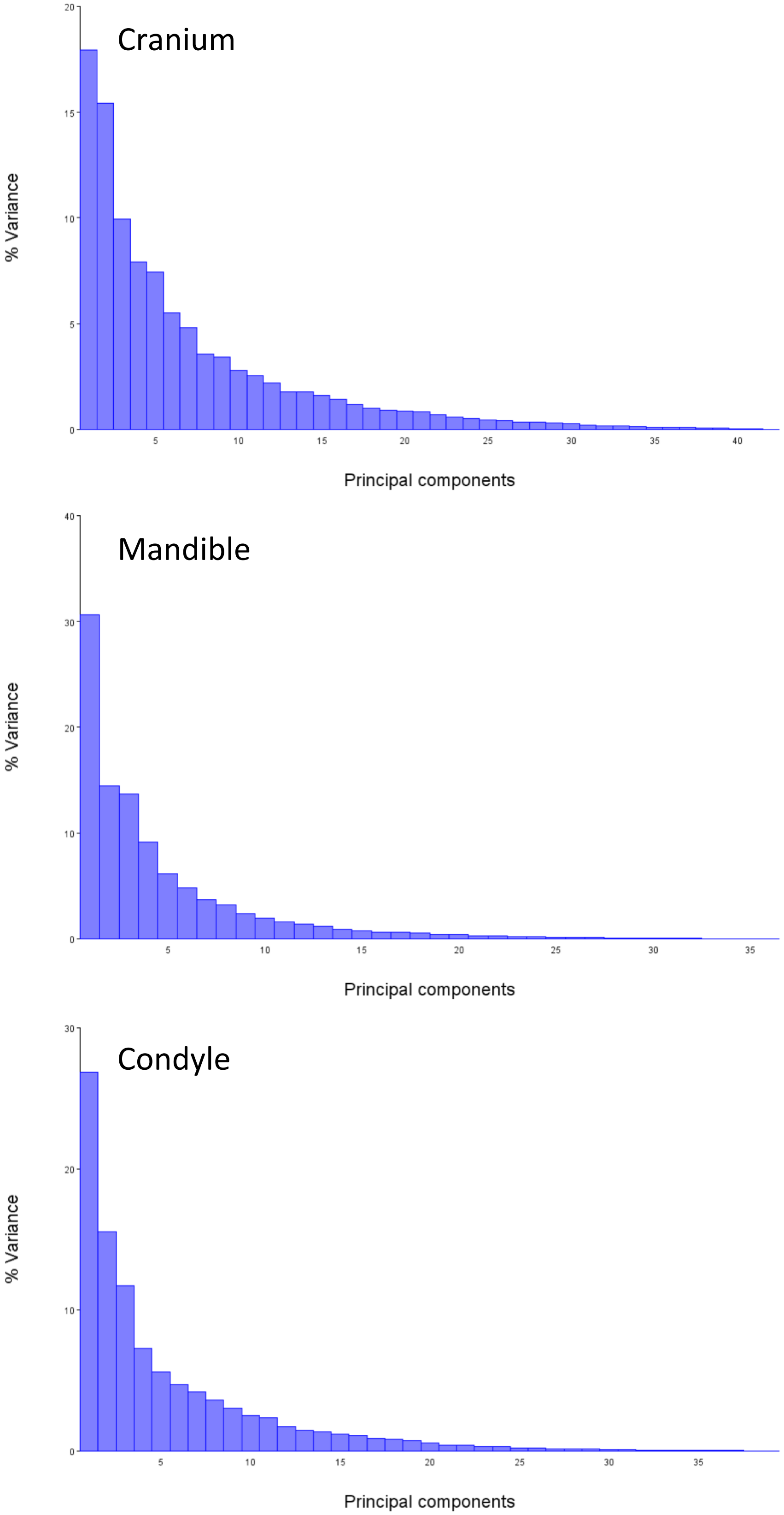

Fig. S4. The percentage of variance in all the principal components of the cranium, mandible, and condyle.

Fig. S5

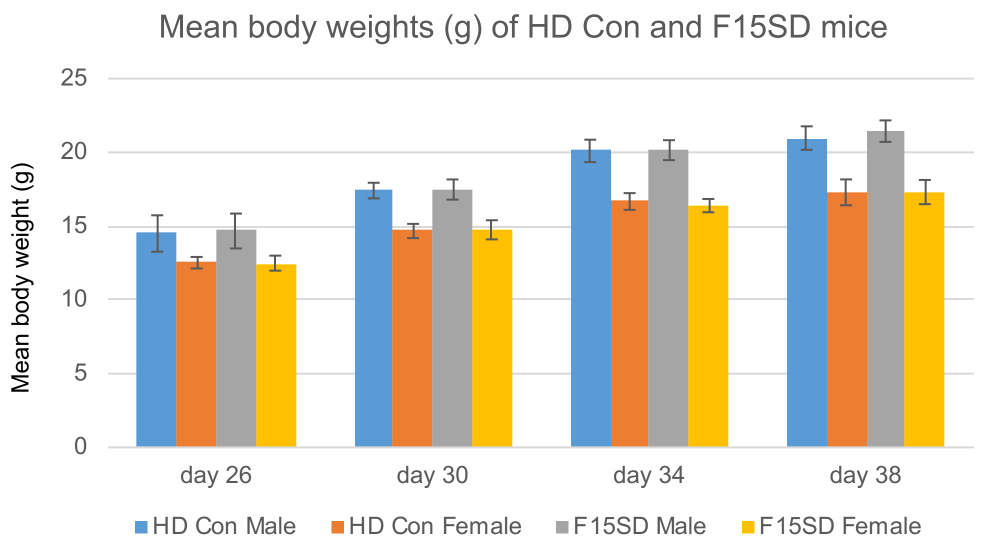
Mean body weights (g) of HD Con and F15SD mice
Mean body weight (g)
25
20
15
10
5
0
day 26
day 30
day 34
day 38
HD Con Male
HD Con Female
F15SD Male
F15SD Female
